# Protective Effect of *Prunus Cerasus* (Sour Cherry) Seed Extract on the Recovery of Ischemia/Reperfusion-Induced Retinal Damage in Zucker Diabetic Fatty Rat

**DOI:** 10.3390/molecules22101782

**Published:** 2017-10-21

**Authors:** Balázs Varga, Dániel Priksz, Nóra Lampé, Mariann Bombicz, Andrea Kurucz, Adrienn Mónika Szabó, Anikó Pósa, Renáta Szabó, Ádám Kemény-Beke, Judit Remenyik, Rudolf Gesztelyi, Béla Juhász

**Affiliations:** 1 Department of Pharmacology and Pharmacotherapy, Faculty of Medicine, University of Debrecen, Debrecen H4032, Hungary; varga.balazs@pharm.unideb.hu (B.V.); priksz.daniel@pharm.unideb.hu (D.P.); lampenori@hotmail.com (N.L.); bombicz.mariann@pharm.unideb.hu (M.B.); kurucz.andrea@pharm.unideb.hu (A.K.); gesztelyi.rudolf@pharm.unideb.hu (R.G.); 2 Department of Internal Medicine, Building C, Faculty of Medicine, University of Debrecen, Debrecen H4032, Hungary; szadrica@freemail.hu; 3 Department of Physiology, Anatomy and Neuroscience, Faculty of Science and Informatics, University of Szeged, Szeged H6726, Hungary; paniko@bio.u-szeged.hu (A.P.); szaborenata88@gmail.com (R.S.); 4 Department of Ophtalmology, Faculty of Medicine, University of Debrecen, Debrecen H4032, Hungary; kemenyba@med.unideb.hu; 5 Institute of Food Technology, Faculty of Agricultural and Food Sciences and Environmental Management, University of Debrecen, Debrecen H4032, Hungary; remenyik@agr.unideb.hu

**Keywords:** *Prunus cerasus*, sour cherry, ischemia-reperfusion, retina, electroretinography, ZDF rat

## Abstract

Among diabetes patients, ophthalmological complications are very frequent. High blood glucose and (consequential) ischemia-reperfusion (I/R) injury contribute significantly to the severity of retinopathies. Diabetic retinopathy is among the leading causes of blindness. Our study demonstrates the effect of sour cherry seed extract (SCSE) on blood glucose and function of the retina with electroretinography (ERG) in a diabetic setting with or without ischemia-reperfusion (I/R) injury in Zucker Diabetic Fatty (ZDF) rats. Our results prove that the SCSE has a retinoprotective effect in diabetic rats: according to ERG measurements, SCSE treatment mitigated the retinal function-damaging effect of diabetes, and proved to be protective in the diabetic eye against ischemia-reperfusion injuries of the retina. Outcomes suggest that the protective effects of SCSE may occur through several pathways, including HO-1 dependent mechanisms. The observation that SCSE treatment decreases blood glucose is also novel. These findings offer the possibility for development of novel therapeutic strategies utilizing this emerging functional food, in particular in the prevention of conditions resulting from high blood glucose or I/R injury, such as deterioration of retinal microcirculation.

## 1. Introduction

Diabetic retinopathy [1], retinal vascular occlusion [2], and glaucoma [3] are some of the many diseases in which ischemia-reperfusion (I/R) injury or high blood glucose levels and the resulting deterioration of tissue microcirculatory capacity are major contributing factors. Dietary intake of phytonutrients—health-enhancing compounds found in plants—substantially reduces the risk of serious diseases and improves the effectiveness of therapy and outcome [4,5,6,7], even in the case of ocular disorders [8]. Previously, the authors of this report carried out experiments with the flavonoid-rich extract of *Prunus cerasus* (sour cherry) seed (SCSE), an emerging functional food [9].

According to the results of a wide spectrum of measurements including GC-MS and HPLC, constituents of SCSE include bio-active compounds such as dihydro-p-coumaric acid, ferrulic acid, caffeic acid, cyanidin, peonidin, squalene, β-tocopherol, γ-sitosterol, and vitamin E, to many of which the sour cherry extract owes its protective, antioxidant capacity [10].

Heme oxygenase 1 (HO-1), also referred to as heat shock protein (HSP) 32, has a key role in the amelioration of oxidative stress-related pathologies, including cardiovascular, lung, neurological, and kidney disorders [11,12,13,14]. Mahmoud et al. [15,16] established a link between SCSE and HO-1: they demonstrated that the extract increases the expression of heme oxygenase 1 besides its antioxidant capacity.

The results of previous work by the authors of this report suggest that sour cherry seed extract may have potential for the prevention and treatment of I/R-associated pathologies, including diabetes-related ocular disease: previously Bak et al. [17], and later Czompa et al. [18], demonstrated the protective effect of SCSE on ischemic-reperfused rat myocardium; Juhasz et al. [4] confirmed a cardioprotective effect in hypercholesterolemic rabbits; and Szabo et al. [19] proved protection in ischemic-reperfused retina. The extract was further studied toxicologically [20], which enabled the use of SCSE even in human studies [21,22]. These results are very promising and make SCSE a potential candidate for future drug development and further studies, such as the present one, to analyze the wide spectrum of effects the extract may exert.

Deterioration of tissue microcirculatory capacity due to ischemia-reperfusion injury or high blood glucose levels causes significantly adverse effects on retinal tissue, however, at the time of this writing, the ability of SCSE to protect the functioning of the retina against ischemia-reperfusion injury in a high blood glucose setting has not been comprehensively investigated.

The present study was conducted to evaluate the effects of sour cherry seed extract (SCSE) on blood sugar levels in Zucker Diabetic Fatty (ZDF) rats. We also wanted to assess the function of the retina in this insulin-intolerant animal model with the help of electroretinography (ERG). A major goal of the experiments was to measure the effect of SCSE treatment on the alteration of retinal function following ischemia-reperfusion injury in such a diabetic setting. We also tried to explore an action–mechanism pathway of the retinoprotective effect of SCSE.

## 2. Results

### 2.1. Fasting Blood Sugar Analysis and Oral Glucose Tolerance Test (OGTT) Results

**Figure 1:**
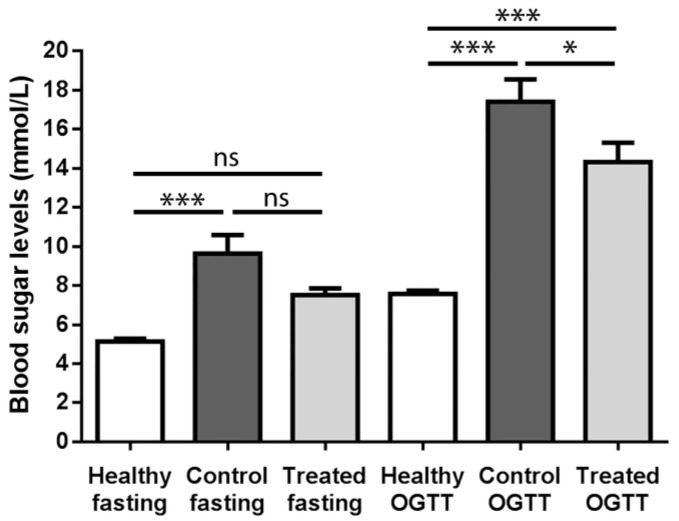
Blood sugar levels of animals before (fasting) and after oral glucose tolerance test (OGTT). Values are mean ± SEM in mmol/L. ns = no significant difference; * *p* < 0.05; *** *p* < 0.001.

Figure 1 shows the blood sugar levels of the three animal groups in the fasting state as well as after oral glucose tolerance testing (OGTT).

The fasting and OGTT blood glucose levels of the healthy animal group (5.137 mmol/L and 7.571 mmol/L, respectively) did not differ significantly from each other, as occurred in the other two diabetic animal groups. The fasting blood glucose level of the control group (9.626 mmol/L) was significantly different from the appropriate value of the healthy group, and apparently differed also from the fasting value of the treated group, however this latter difference was statistically not significant. At the same time, the fasting blood glucose level of the treated group (7.510 mmol/L) was statistically not different from the fasting results of the healthy animals either. In the case of OGTT blood sugar values, both diabetic groups differed significantly from healthy group values: the mean value of the control group was 17.41 mmol/L, while that of the treated group was 14.330 mmol/L. The difference between the control and treated groups was also significant (*p* < 0.05).

### 2.2. Electroretinography

**Figure 2:**
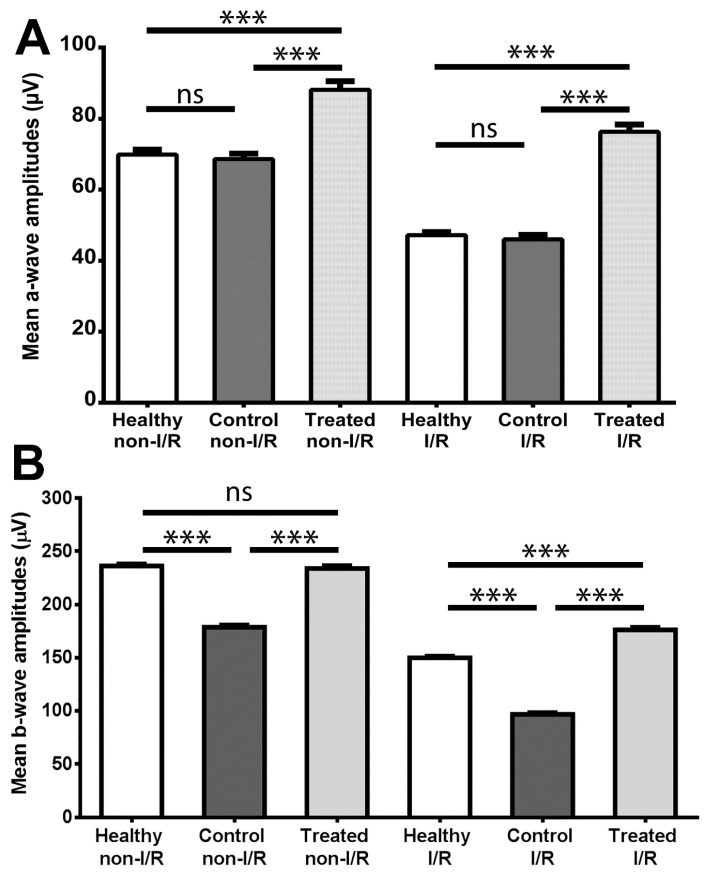
Electroretinographical results. (**A**) Mean a-wave amplitudes; (**B**) Mean b-wave amplitudes; Mean retinal a- and b-wave amplitudes elicited by light flashes were measured both in non-ischemic/reperfused (non-I/R) and in ischemic-reperfused eyes (I/R) of the different groups. Values are mean ± SEM in micro-Volts (µV). ns = no significant difference; *** *p* < 0.001.

The outcome of ERG measurements is shown in Figure 2, which demonstrates mean amplitudes of a- and b-waves (Figure 2A,B, respectively) of electroretinograms in the three animal groups.

By comparing the non-IR data, it can be seen that the SCSE treatment significantly increased the mean amplitudes of a- and b-waves relative to the control group (88.11 μV vs. 68.61 μV for the a-waves and 233.9 μV vs. 178.7 μV for the b-waves; *p* < 0.001 for both comparisons); furthermore, the mean amplitude of b-waves of the treated group did not differ significantly from the same values of the healthy group (233.9 μV vs. 236.2 μV). In the case of a-waves, the former mentioned comparison results in a significant difference between treated and healthy non-I/R groups, with the treated group being higher (88.11 μV vs. 69.85 μV).

Regarding the IR values, it can be concluded that a significant difference can be seen between the b-wave mean amplitudes of the control group and that of the healthy animals (96.83 μV vs. 149.9 μV; control vs. healthy; *p* < 0.001), while the treatment increased these values significantly (176.2 μV; *p* < 0.001 vs. control). It should be highlighted that the IR b-wave results of the treated group also proved to be significantly better than the IR b-wave values of the healthy group (treated vs. healthy; *p* < 0.001). Similarly, sour cherry seed extract provided better a-wave amplitudes after ischemia-reperfusion as compared either with healthy or control groups (76.28 μV vs. 47.15 μV or 45.98 μV; treated vs. healthy or control; *p* < 0.001 in both comparisons).

Representative electroretinograms of each experimental group are shown in Supplementary Materials Figure S1.

### 2.3. Measurement of Heme Oxygenase Concentration

**Figure 3:**
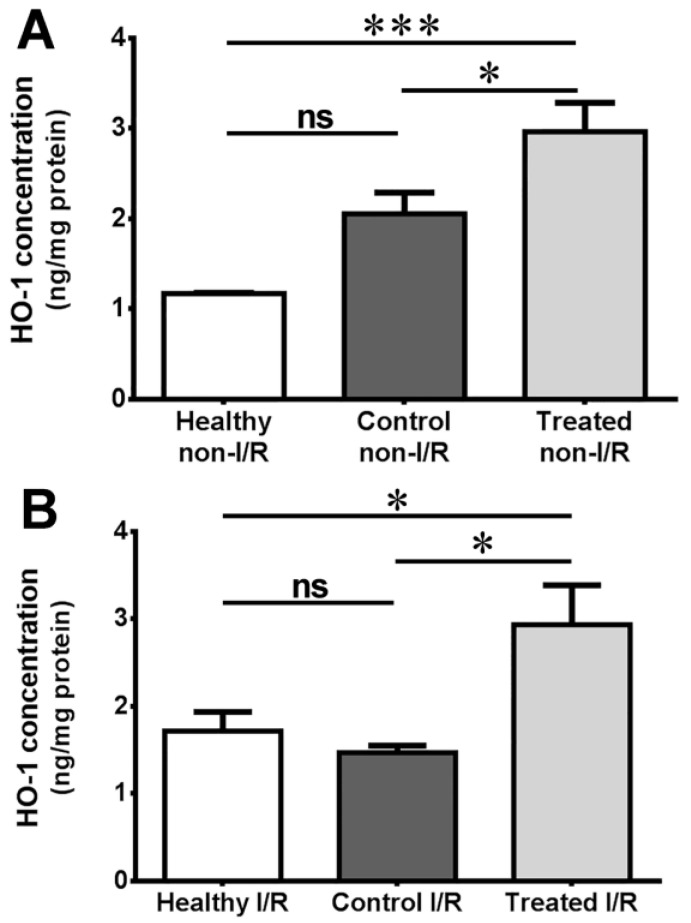
Results of heme oxygenase 1 concentration measurement. (**A**) Non-ischemic/reperfused eyes; (**B**) Ischemic and reperfused eyes. Carried out from the removed bulbi of animals of the different groups, this measurement estimates the amount of heme oxygenase 1 enzyme (ng) per mg (total) protein. Values are mean ± SEM in ng/mg; ns = no significant difference; * *p* < 0.05; *** *p* < 0.001.

The outcome of heme oxygenase concentration measurement of ocular tissue from rats of the different groups is provided in Figure 3.

As shown in this figure, tissue concentration of the enzyme was statistically the same in the healthy and control groups, both pre-ischemically and after ischemia (1.170 vs. 1.115 for non-I/R and 1.717 vs. 1.469 for I/R, respectively). In the case of non-I/R eyes, a significant boost in expression is seen in the SCSE-treated group compared to both the healthy and the control groups (2.963; *p* < 0.05 vs. control and *p* < 0.001 vs. healthy). The elevation in ischemia protein expression in the healthy group is the reason why the healthy vs. treated comparison lost its former strength, nevertheless, the mean concentration value of the treated group was still significantly higher compared with the healthy group (2.934; *p* < 0.05 vs. healthy/control).

### 2.4. Histology Results

**Figure 4:**
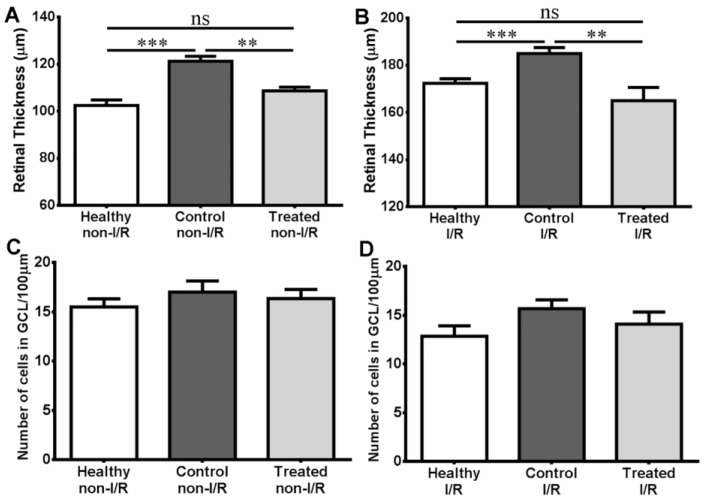
Histology results. (**A**) Retinal thickness of non-ischemic-reperfused eyes of the different groups; (**B**) Retinal thickness of ischemic-reperfused eyes of the different groups; (**C**) Number of cells in ganglion cell layer per unit distance (100 μm) in non-ischemic-reperfused eyes of the different groups; (**D**) Average number of cells in ganglion cell layer per unit distance (100 μm) in ischemic-reperfused eyes of the different groups. Values are mean ± SEM; ns = no significant difference; ** *p* < 0.01; *** *p* < 0.001. In panels C and D, no significant differences are seen.

Quantitative histology results of ocular tissue from rats of the different groups are provided in Figure 4.

As demonstrated in this figure, retinas of the control non-I/R group are significantly thicker than in healthy animals (121.3 μm vs. 102.4 μm, *p* < 0.001), while the retinal thickness of the treated group (108.7 μm) does not differ from the healthy value, but does from control non-I/R (*p* < 0.01). Similar comparisons can be seen after ischemia-reperfusion (172.3 μm vs. 185.0 μm vs. 165.0 μm). In the case of average ganglion cell numbers per unit distance (100 μm), no significant differences were seen between the different groups either before (15.50 vs. 17.00 vs. 16.33, respectively), or after ischemia-reperfusion (12.83 vs. 13.67 vs. 11.35).

Hematoxylin-eosin-dyed representative sections of retinae from bulbi of the different groups are shown in Supplementary Materials Figure S2.

## 3. Discussion

Diabetes mellitus is a metabolic disease with an impaired carbo-hydrate turnover, characterized by a decreased rate of insulin secretion and reduced insulin sensitivity of cells expressing insulin receptors. It is well known that, among diabetic patients, ophthalmologic complications are very common: after age-related macular degeneration, diabetic retinopathy is the second leading cause of blindness in Hungary [23].

In the present study, the effect of the flavonoid-rich extract of the *Prunus cerasus* (sour cherry) seed on the blood glucose levels of the Zucker diabetic fatty rat—a glucose intolerant animal model—was examined. With the help of electroretinography, the change in retinal function related to diabetes, IR injury, and their combination, with or without SCSE administration, was investigated. Furthermore, the change of HO-concentration in the retinal tissues of the different groups was quantified as well. In addition, we carried out quantitative analyses on histological sections, namely measuring retinal thickness and counting of cells in the ganglion cell layer (GCL) per unit distance in the formerly mentioned settings.

According to our OGTT measurements, sour cherry seed extract showed a modest, but significant blood sugar lowering effect: blood glucose levels of the diabetic animal groups were higher than the values of the healthy animals, however, values of the treated group after oral glucose load were improved compared with control (Figure 1). The above-mentioned findings may be explained by the fact that long before the actual (fasting) blood glucose elevation and the appearance of clinical diabetes, glucose intolerance develops on the basis of insulin resistance, a condition which has been recognized as the most significant predictor of further development of type 2 diabetes mellitus [24].

Several studies have shown that phytocompounds found in fruits and vegetables have beneficial health effects like prevention of cancer, cardiovascular diseases, and obesity [25]. Most recently, Lachin et al. [26] published a paper on the effect of different cherries on diabetes with many promising results: e.g., yellow cherry, sweet cherry, or tart/sour cherry fruit showed significant blood sugar lowering effects. The data of our experiments contribute to this finding with a novel result, that an extract made of sour cherry seed may also be modestly antidiabetic.

Data from the electroretinographical measurements provide insight into the damaging effects of diabetes mellitus on retinal function (when comparing the non-IR results of healthy and control animals), and allow easy comparison of functioning of IR-injured and non-IR retina in the animal groups (Figure 2). A major finding of this current report is the significant improvement in the electrophysiological functions of photoreceptors and retinal cells post-synaptic to photoreceptors (i.e., cells of the inner nuclear layer (INL)) as seen on non-IR electroretinograms of the SCSE-administered group compared with control. Despite the high blood glucose levels, retinal function of non-IR eyes of animals in the treated group was at least similar to that of the healthy group, which is also a major finding. This means that SCSE treatment was able to prevent the retina-damaging effect of diabetes mellitus on the functional level: both photoreceptors and post-receptoral on pathway cells, including rod bipolar cells, remain highly active despite the deteriorative effect of hyperglycaemia. Such retinoprotective effects were demonstrated by other authors using treatment with resveratrol, an active agent also of herbal origin [27], or with peptides such as GLP-1 analogue exenatide or liraglutide [28,29].

A number of ocular diseases have been associated with retinal ischemia-reperfusion injury including diabetic retinopathy [30]. A simple method of provoking IR injury used in the present study offered insight to the retina-damaging consequences of diabetes. Upon investigating the IR values on our electroretinograms, it can be concluded that, while a significant deterioration in retinal function can be seen in the control group compared with healthy animals, significant functional improvement was demonstrated in the treated group. Furthermore, the SCSE-treated group showed significantly better retinal function compared with the healthy animal group. This novel result supports the hypothesis that SCSE may have capacity in preventing IR-induced retinal damage at the functional level even in a diabetic setting. In non-diabetic rat models, our workgroup already published intriguing results on the effect of SCSE against IR-injury [19] and also with other treatments including alpha-MSH or PACAP [14,31]. Similarly, further potential possibilities can be found in the scientific literature for treatment or prevention of IR-injured retina in diabetic retinopathy, such as with the GLP-1 peptide analogue, exeantide [32].

The HO-1 enzyme may play an important role in the retinoprotective effect of sour cherry seed extract in a diabetic setting, as could be concluded from heme oxygenase 1 (HO-1) concentration measurements presented here (Figure 3A). Elevated blood sugar levels may imply a stress for the retinal tissue, as the level of this well-known stress protein—although not significantly, but still—increases in the control group as compared with healthy, low blood glucose animals. The sour cherry seed extract further elevated the level of this protective enzyme, which may contribute to the hypoglycemic activity of the extract. Similar elevated heme oxygenase enzyme concentration after sour cherry seed extract treatment was seen formerly in conventional, i.e., non-diabetic animal models in other tissues [4,16] and even in retina as well [19]. A novel finding of this current report is that the capacity of SCSE in elevating HO-1 levels was confirmed in a diabetic setting.

Ischemia-reperfusion damages the retina [14] as well as other tissues [12]. Based on our present investigation the level of heme oxygenase may increase upon such injury (Figure 3B: healthy I/R group). In the diabetic animals (control I/R group), however, the harmful effect of diabetes and ischemia-reperfusion were added together, which may be associated with the decrease of heme oxygenase concentration. Despite the deteriorating effects of a high-glucose setting, sour cherry seed treatment generated a higher HO-1 concentration as compared with the healthy group, which is a novel result, and which may be the reason behind the protective effect of the extract against ischemia-reperfusion injury. According to our knowledge, this report is the first to suggest a retinoprotective effect of SCSE on diabetic animals, which was connected to heme oxygenase overexpression. Similar mechanisms of effect are not unheard of among herbal medicines: retinoprotective effects of blueberry anthocyanins are also mediated through the Nrf2/heme oxygenase signaling pathway [8]. Based on our former results, and those of other authors, heme oxygenase 1 is a highly potent protective agent against ischemia-reperfusion injury [13,19,33], which was also confirmed and corroborated in our present study. Nonetheless, it is necessary to investigate other possible mechanisms to facilitate deeper understanding of the exact effects of SCSE. Further avenues of future experiments may include exploring possible involvement of hypoxia-induced factor 1 alpha (HIF1-α), HIF2-α, or different heat shock proteins such as hsp60 or 90.

Sour cherry seed extract was able to counteract the edema-inducing effect of diabetes as seen from the comparison of treated non-I/R and control non-I/R retinal thickness values (Figure 4A). Ischemia-reperfusion injury may also cause retinal edema [34], an effect compensated by SCSE treatment based on retinal thickness values of ischemic-reperfused retina tissue samples (Figure 4B). These novel results are not individual among herbal agents: such a protective effect was demonstrated with other herbal treatments, such as *Lycium barbarum* [35,36], ginsenosides [37] or Flos puerariae [38].

Ganglion cell numbers did not change significantly according to our results (Figure 4C,D). The reason behind this may be the relatively short time interval between the ischemia and tissue sampling: the time course of ischemia-reperfusion injury starts with initial retinal edema, followed by structural degenerative changes which need more time to develop, i.e., a consequential cell death, e.g., a decrease in number of ganglion cells [34].

All things considered, according to our data presented here, SCSE may have the capacity to alleviate the damaging effect of high blood glucose and protect the retina from I/R injuries in diabetic conditions. Although this extract will not be the first choice for lowering blood glucose levels, nevertheless there are many other drugs for that purpose to choose from, and an agent can indeed have a potent retinoprotective effect in diabetic retinopathy without being effective against hyperglycaemia [29]. Formulation of new drugs from such synergistic agents—be it of herbal origin or not—can result in the successful treatment of an unmanageable disease such as diabetic retinopathy. For this reason, our observations also bear a high potential from a clinical point of view. Development of a future therapy based on SCSE may possess pharmaceutical significance as well.

## 4. Materials and Methods 

### 4.1. Animals and Groups

At the start of the present study, 6-week-old male Zucker Diabetic Fatty (ZDF-Leprfa/Crl) rats and their control (lean phenotype) were purchased from Charles River Laboratories International, Inc. (Wilmington, MA, USA). All animals received humane care in compliance with the ARVO Statement for the Use of Animals in Ophthalmic and Vision Research and the NIH guidelines. All of the protocols used in the present study were approved by the Institutional Animal Care Committee of University of Debrecen in Debrecen, Hungary (18/2013/DE MÁB). The animals were fed a Purina 5008 diet ad libitum for 50 days, and had free access to water.

Rats were separated into three groups (n = 6 in each group): a control diabetic group, a treated diabetic group, and a healthy group. The treated group was gavaged with 30 mg/kg SCSE daily, while the control group was gavaged with the vehicle only (methyl-cellulose mucilage) according to former protocol [17].

### 4.2. Ocular Ischemia and Reperfusion (I/R)

At the end of the 50-day period, rats were anaesthetized with an intramuscular ketamin/xylazine (50/5 mg/kg) injection. The eyes of the animals were locally anaesthetized with 0.4% oxybuprocain eyedrops as well (Humacain, Teva, Debrecen, Hungary). Subsequently, the retinal artery of the left eye of each animal was surgically occluded to cut off the blood supply using a protocol previously applied by the authors [14,34]. A silk suture thread guided through a polyethylene cannula was placed loosely behind the eyeball around the optic nerve, central retinal artery, ciliary arteries, and the retrobulbar connective tissue. By pulling the suture and pressing the tube against the surface of the optic nerve this traction-type occluder induced retinal artery blockage, which could be maintained for the required length of time (1 h). Ischemia was verified macroscopically with a 120-D lens. During ischemia the eye of the rats was covered with sterile gauze. To prevent the drying out of the cornea, carbomer-based eye gel was also used (Vidisic, Bausch&Lomb, Berlin, Germany). Reperfusion of the retinal tissue was accomplished by post-ischemic release of the occluder, allowing resumption of blood flow through the retinal artery.

### 4.3. Electroretinography (ERG)

After 24 h of reperfusion, animals were prepared for electroretinographic measurements by anesthesia with an intramuscular injection of 50/5 mg/kg of ketamin/xylazine. The pupils of each animal were dilated with 0.5% cyclopentolate hydrochloride (Humapent, Teva, Debrecen, Hungary). Five silver electrodes were used for each measurement as follows. At the corneal surfaces, retinal signals were analyzed using two measuring electrodes (one on each eye), inserted so as to avoid scleral damage or corneal perforation. Reference electrodes were positioned on the earlobes of each animal (one on each earlobe), with the main ground electrode at the glabella. Effective electrical contacts and protection of eyes from dehydration was provided by carbomer-based eye gel (Vidisic, Bausch&Lomb, Berlin, Germany). The ERG measurements were carried out in darkness, after a dark adaptation period (20 min) based on the guidelines of the International Society for Clinical Electrophysiology of Vision (ISCEV) [39]. For stimulation of the retina, the eyes were illuminated with a stroboscope (20 cd/m^2^, 0.5 Hz) [39]. Electrical signals, i.e., retinal responses to light flashes, passed through an amplifier and an analog–digital converter (Bridge Amp and PowerLab, ADInstruments, Sydney, Australia), displayed on a PC monitor, recorded and analyzed using PowerLab Chart software (Version 5.2.2., ADInstruments, Sydney, Australia).

The outcome of the electroretinography experiments formerly conducted by the authors demonstrated that the aforementioned experimental strategy provides reproducible, cost-effective data on retinal function, closely correlated to the survival of the retinal cells [14,31].

### 4.4. Oral Glucose Tolerancy Test (OGTT)

Oral glucose tolerancy testing was carried out two times during the experiment: first, when the animals got involved in the experiment, and second, at the end of the 50 day treatment period. Twelve hours before the test, food was withdrawn from the animals. During the test, fasting glucose levels were obtained first, then 1 g/mL glucose solution was prepared with water, which then—after heating to body temperature—was gavaged into the stomach of each animal in a dose of 3 g/kg. One hour after the glucose-load, the blood sugar levels of the animals were measured again. The measurement was carried out using an AccuChek Active blood sugar monitor, which was formerly calibrated by the blood test laboratory of our clinic.

### 4.5. Measurement of Heme Oxygenase Concentration

After the ERG measurements, the eyeballs of the animals were removed for further analysis. Half of the enucleated bulbi were suspended in a homogenization buffer composed of *N*-2-hydroxyethylpiperazine-2-ethanesulfonic acid (HEPES) 10 mM, sucrose 32 mM, dithiotreitol (DTT) 1 mM, ethylenediaminetetraacetic acid disodium salt dihydrate (EDTA) 0.1 mM, soybean trypsin inhibitor 10 μg/mL, Leupeptin 10 μg/mL, Aprotinin 2 μg/mL; pH 7.4 (Sigma-Aldrich, St. Louis, MO, USA). The supernatant was collected by a 30 min centrifugation of the homogenate at 20,000× *g* at 4 °C. The HO-1 content was determined by enzyme-linked immunosorbent assays (ELISA) according to the manufacturer’s directions (Sunred Biotechnology Company, Shanghai, China). Optical density was measured at 450 nm (Benchmark Microplate reader; Bio-Rad, Hercules, CA, USA) and the values were expressed as ng/mg protein.

### 4.6. Processing for Examination by Light-Microscopy

The other half of the enucleated bulbi were processed according to the following general protocol: the vitreum of each eye was removed and the bulbs were fixed in Bouin-solution, alcohol-dehydrated, paraffinized, and were processed into 7 μm sagittal sections, which were then dyed with hematoxylin-eosin (HE) and examined by light microscopy.

Average retinal thickness was measured between the inner limiting membrane and the retinal pigment epithel (ILM-RPE) and expressed in micrometers using a manual scale on each glass slide.

The number of cells in the ganglion cell layer per unit distance (100 μm) was also counted with the help of the same manual scale on each glass slide.

For the quantitative analyses, 6 eyes per group and 6 sections per eye were analyzed.

### 4.7. Statistical Analysis

A one-way analysis of variance with a Tukey or Newman–Keuls post-test was used for Gaussian data results from the D’Agostino & Pearson omnibus normality test. Data with non-parametric distribution were analyzed using the Kruskal–Wallis test along with the Dunns post-test. Figures are represented with standard error of the mean (SEM).

## Supplementary Materials

The following are available online. Figure S1: Representative electroretinograms for each experimental group, Figure S2: Hematoxylin-eosin-dyed representative sections of retinae from bulbi of the different groups. (**a**): healthy non-I/R; (**b**): control non-I/R; (**c**): treated non-I/R; (**d**): healthy I/R; (**e**): control I/R; (**f**): treated I/R.

## Abbreviations

The following abbreviations are used in this manuscript:

ZDF rats	Zucker Diabetic Fatty rats
SCSE	Sour cherry seed extract
ERG	Electroretinography
HO-1	heme oxygenase-1
I/R	Ischemia/reperfusion
