# Supplementary material for: Protective Effect of Prunus Cerasus (Sour Cherry) Seed Extract on the Recovery of Ischemia/Reperfusion-Induced Retinal Damage in Zucker Diabetic Fatty Rat
Source: Molecules. 2017 Oct 21;22(10):1782. doi: 10.3390/molecules22101782 (PMC6151469; doi:10.3390/molecules22101782)
Supplement: Supplementary file 1 [file molecules-22-01782-s001.pdf]

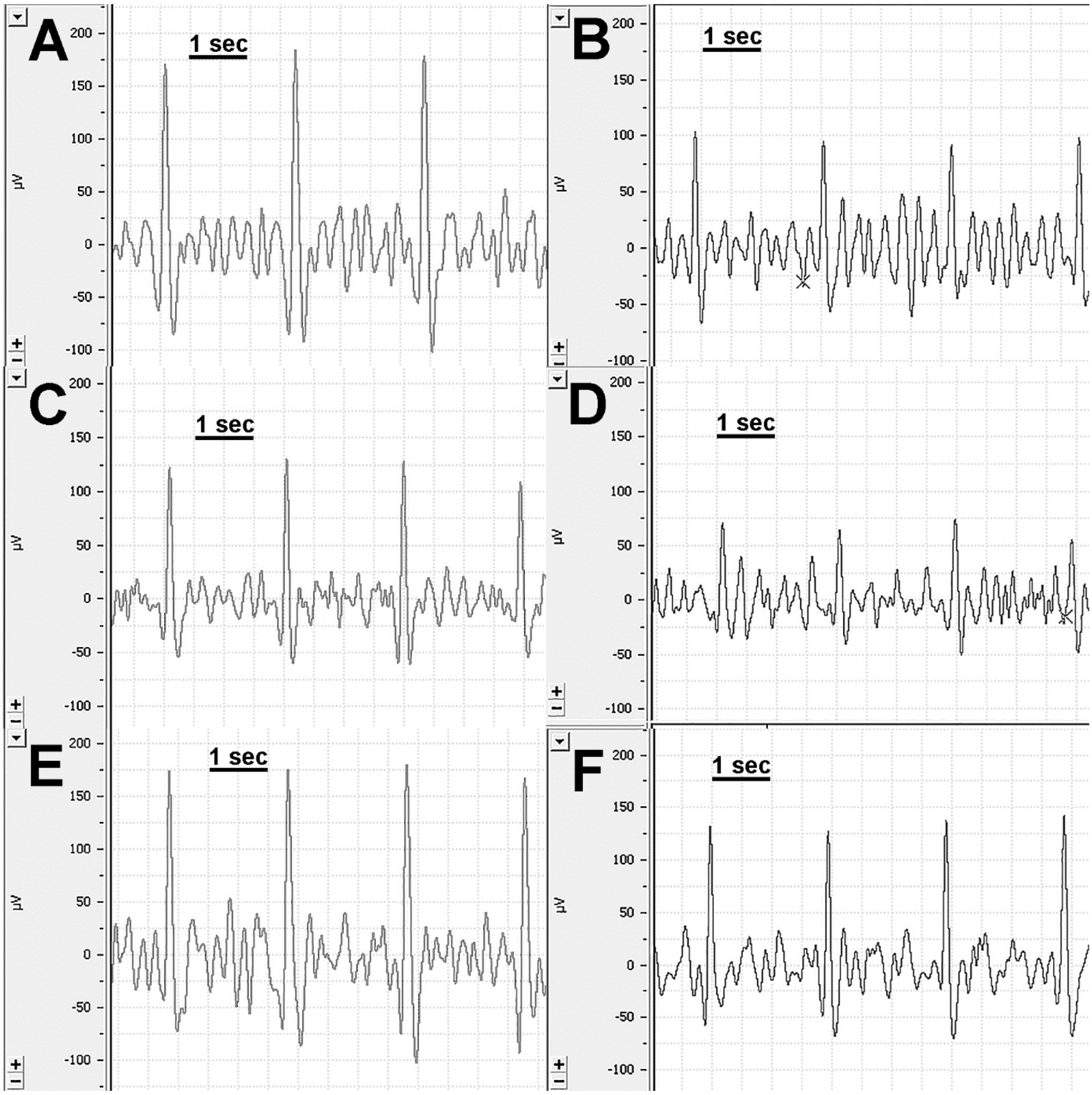

Supplementary Figure 1. Representative electroretinograms for each experimental group.

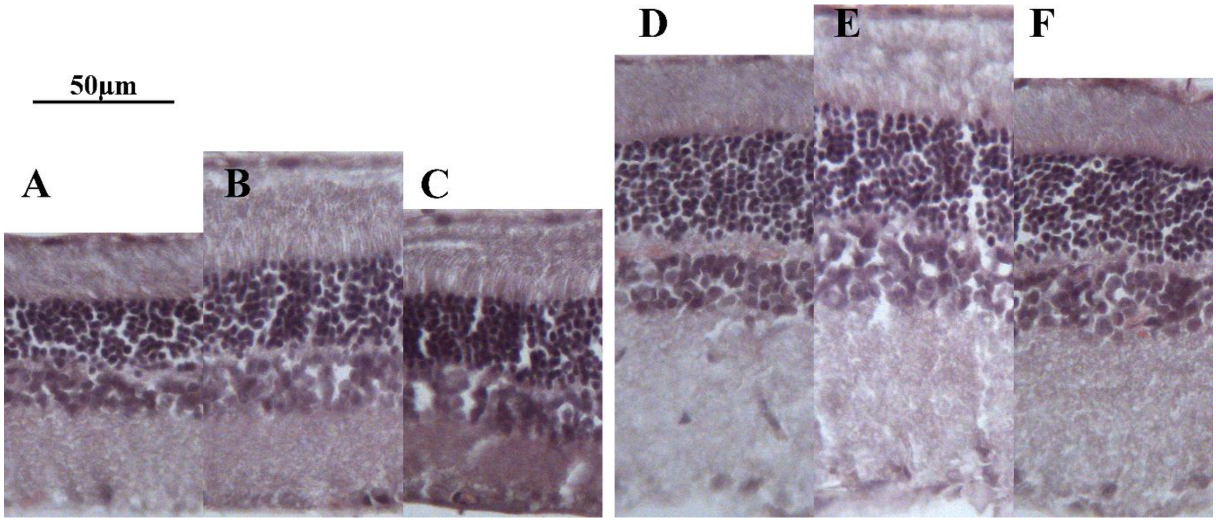

Supplementary Figure 2. Hematoxylin-eosin-dyed representative sections of retinae from bulbi of the different groups. (**a**): healthy non-I/R; (**b**): control non-I/R; (**c**): treated non-I/R; (**d**): healthy I/R; (**e**): control I/R; (**f**): treated I/R
